# Dynamic calcium–potassium balancing by KCNMA1 preserves the epithelial/mesenchymal hybrid state and modulates therapy response in ovarian cancer

**DOI:** 10.1038/s41420-026-03189-6

**Published:** 2026-06-04

**Authors:** Tereza Buchtova, Jirina Bartkova, Tatsuro Yamamoto, Marie Lund Bay, Allan Jensen, Susanne Krüger Kjær, Tuula Kallunki, Jiri Bartek, Robert Strauss

**Affiliations:** 1 Genome Integrity Group, Danish Cancer Institute, Danish Cancer Society, Copenhagen, Denmark; 2 Department of Medical Biochemistry and Biophysics, Division of Genome Biology, Science for Laboratory, Karolinska Institute, Solna, Sweden; 3 Cancer Invasion and Resistance Group, Danish Cancer Institute, Danish Cancer Society, Copenhagen, Denmark; 4 Virus, Lifestyle and Genes Group, Danish Cancer Institute, Danish Cancer Society, Copenhagen, Denmark; 5 Dept. of Gynecology, Copenhagen University Hospital, Rigshospitalet, Copenhagen, Denmark; 6 Translational and Clinical Pharmacology, Drug Design and Pharmacology, University of Copenhagen, Copenhagen, Denmark

**Keywords:** Ovarian cancer, Cancer stem cells, Chemotherapy

## Abstract

Tumor cell plasticity and stemness fuel treatment resistance and cancer evolution into often incurable, metastatic terminal disease. To better understand these fundamental aspects of tumorigenesis, here we examine a potential role of KCNMA1, a calcium-activated potassium channel that impacts cell (patho)physiology through membrane functions, in regulating ovarian cancer cell behavior, including plasticity, proliferation, mobility, and response to treatment. Pharmacological activation of KCNMA1 promoted differentiation, while channel blocking induced dedifferentiation and enhanced dissemination potential. Cyclical activation and inhibition potentiated the epithelial/mesenchymal hybrid cell state prone to stemness. KCNMA1 overexpression combined with low-dose channel blockade supported three-dimensional tumor growth. Mechanistically, we found that the balance between cytosolic calcium and potassium, in addition to their absolute levels, governed the observed changes. Our findings support a model in which KCNMA1-mediated regulation of potassium buffers fluctuating calcium signals that drive phenotypic plasticity, thereby stabilizing the epithelial/mesenchymal hybrid state. In addition, KCNMA1 modulation sensitized ovarian cancer cells to standard-of-care chemotherapeutics. Together, this work provides new insights into the role of KCNMA1 and calcium/potassium homeostasis in ovarian cancer cell adaptability, with implications for treatment.

**Figure:**
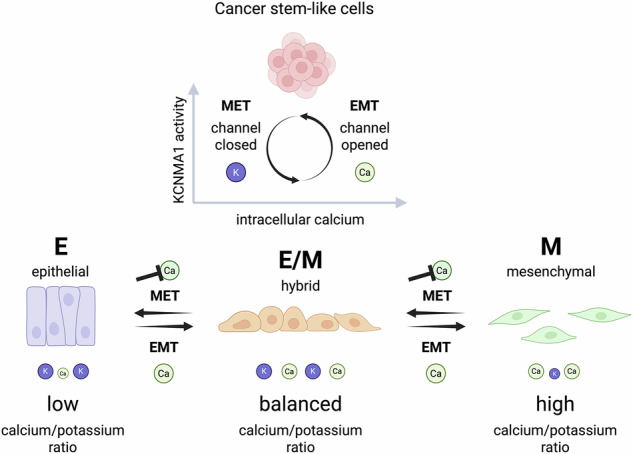

## Introduction

Resistance to standard chemotherapy remains a major obstacle in oncology, and numerous cellular mechanisms contribute to treatment failure. A key driver of treatment failure is cell plasticity, which includes transitions between epithelial and mesenchymal phenotypes that enable cancer cells to resist therapy-induced stress, migrate, and regenerate tumors [1]. Epithelial cells have a polarized cell shape and are characterized by prominent intercellular tight and adherens junctions featuring E-cadherin expression, whereas mesenchymal cells lack this structural polarity and display a more spindle-like morphology with reduced cell-cell adhesion [2]. Importantly, changes between these phenotypes happen gradually, leading to a transitional epithelial/mesenchymal (E/M) hybrid state, in which cells feature both epithelial and mesenchymal traits. We and others have proposed that this hybrid state is not only a transient intermediate but also contains a subpopulation of metastable, self-sustaining cells with stem-like behavior and high adaptability [2–5]. Plastic transitions between epithelial, E/M hybrid, and mesenchymal states are orchestrated by multiple intracellular and extracellular cues, including growth factors, redox signals, and ion fluxes, and are further shaped by the tumor microenvironment [2, 6]. Therapeutic interventions may also lead to epithelial-mesenchymal transition (EMT) and associated complex gene expression as well as cell signaling changes that promote therapy resistance mediated by increased drug efflux, aberrantly enhanced DNA damage repair, apoptosis resistance and/or establishment of an immunosuppressive environment [3, 6, 7].

A clinically relevant model of such plasticity is ovarian cancer, the most lethal gynecological malignancy, with over 320,000 new cases reported in 2022 [8]. It is a heterogeneous disease, subdivided into five clinically and genetically distinct types among the epithelial tumors of the ovary, where the high-grade serous type is the most common. Survival rates vary geographically and with stage. Data from the US show that the 5-year relative survival for localized ovarian cancer is 91.7%, whereas that of late-stage disease is around 30% [9]. For stage 1 ovarian cancer, chemotherapy is recommended after surgery for the majority, and for all stage 2–4 cases, surgery and chemotherapy are recommended. Unfortunately, due to a lack of symptoms in the early stages, the majority of women (>80%) are diagnosed at a late stage, which leads to poor survival despite initial response to standard paclitaxel and carboplatin combination therapy [10]. Most of these tumors are now thought to originate from the fallopian tube epithelium, with serous tubal intraepithelial carcinoma as a precursor lesion [11, 12]. In ovarian, but also other cancer types, a key driver of tumor plasticity is a subpopulation of cancer stem-like cells with an E/M hybrid phenotype distinguishable by protein markers such as CD133, CD44, and cytoplasm-retained E-cadherin. These cancer stem-like cells can self-renew, give rise to new tumors, generate phenotypically diverse progeny, and are associated with treatment resistance and poor patient outcome [5, 13–15]. Understanding the mechanisms that maintain their E/M hybrid identity is, therefore, central to overcoming plasticity and improving cancer therapy.

Calcium and potassium ions regulate numerous processes in cell physiology. Calcium-regulated signaling is known to control growth, metabolism, gene expression, motility, as well as apoptosis [16]. Maintenance of calcium homeostasis is ensured by dynamic calcium pumping among organelles as well as between the intracellular and extracellular space, with interlinked calcium-sensing mechanisms and feedback loops [17]. While extracellular calcium is required to stabilize epithelial junction proteins such as E-cadherin [18], elevation of cytosolic calcium triggers rapid cortical actin remodeling [19] and E-cadherin cleavage [20]. Dedicated calcium channels, which act in response to stimuli such as membrane depolarization or calcium depletion from organelles, can raise cytosolic calcium and induce EMT by activating transcription factors including NFAT, Snail and ZEB1 [21, 22]. In contrast, continuous potassium efflux and subsequent membrane hyperpolarization drive epithelial differentiation [23]. Intracellular potassium is thereby critically needed for E-cadherin recycling [24] and acts as a functional component of the Na/K-ATPase that in turn facilitates potassium reentry to drive the formation of epithelial cell junctions and cell polarity [25].

Ion channel activity has recently emerged as a key regulator of cancer cell state transitions rather than merely a permissive component of cellular physiology. Coordinated calcium influx and potassium flux are increasingly recognized to influence stemness, epithelial–mesenchymal plasticity and tolerance to therapeutic stress [26]. Large-conductance, calcium- and voltage-activated potassium (BK) channels provide a direct molecular link between calcium influx and potassium efflux. The channel’s α-subunit, encoded by KCNMA1, opens in response to cytosolic calcium spikes and membrane depolarization, and allows for potassium outflow that repolarizes the membrane and limits further calcium entry. Through this feedback loop, BK channels act as fine-tuned regulators of intracellular calcium homeostasis and excitability [27]. In healthy tissues, KCNMA1 expression is highest in the brain and gastrointestinal tract and is also detected in the epithelium of the reproductive tissue [28, 29]. Its activity is modulated by phosphorylation, lipid interactions, and ubiquitin-mediated turnover via the FBXW7 ligase [30, 31].

BK channels have essential physiological functions in controlling vascular tone, smooth-muscle contraction, and neuronal excitability [32]. Dysregulation of KCNMA1 has been associated with neurological and cardiovascular disorders as well as several cancers [30–33]. Intriguingly, both oncogenic and tumor-suppressive roles have been reported for KCNMA1: KCNMA1 overexpression promotes invasion and stemness in glioblastoma and breast cancer, but correlates with a favorable outcome and differentiation in gastric carcinoma [34–36]. These discrepancies suggest that the functional impact of KCNMA1 depends on tissue context and possibly on the direction of calcium/potassium flux it mediates.

Despite emerging evidence that ion channels shape cancer cell behavior, how KCNMA1-mediated ionic flux influences epithelial/mesenchymal plasticity, stemness and therapy response remains poorly understood. In the present work, we investigate the role of KCNMA1 in human ovarian cancer models to determine whether and how this channel governs epithelial cell plasticity. Through transcriptomic correlation, pharmacological and genetic modulation, as well as ion-sensitive imaging, we find that KCNMA1 activity enables epithelial cancer cells to buffer EMT or the reverse mesenchymal-epithelial transition (MET) through controlled calcium/potassium exchange. Our findings demonstrate that ion balancing thereby stabilizes the stemness-associated E/M-hybrid state and impacts therapy response in ovarian carcinoma.

## Results

### KCNMA1 channel activity impacts cancer cell differentiation and plasticity

**Fig. 1:**
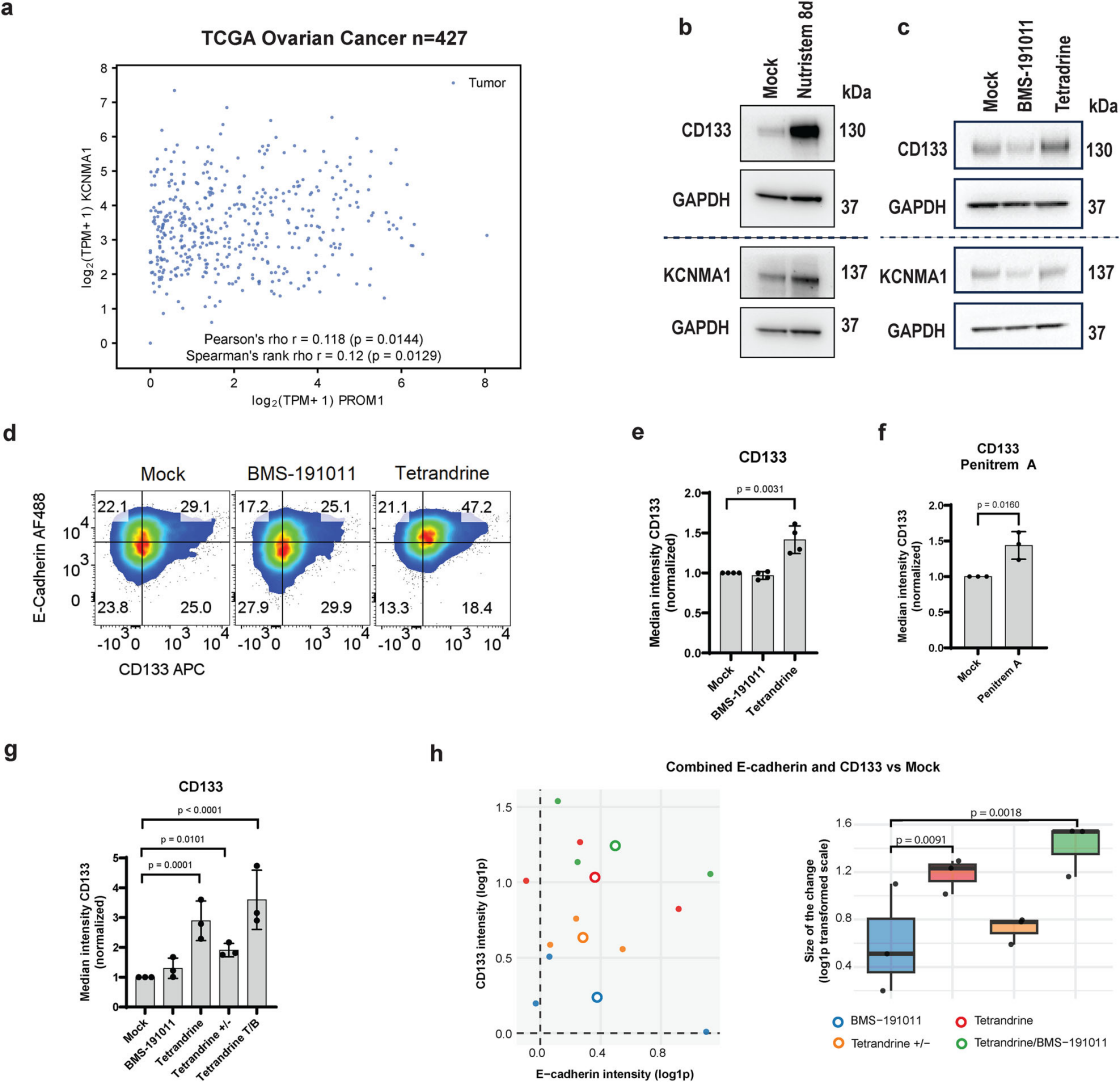
KCNMA1 expression correlates with CD133, and its activity oscillation elevates CD133 and E-cadherin levels. **a** Scatter plot visualizes correlation between *PROM1* and *KCNMA1* in ovarian cancer. Expression values are shown as log2(TPM + 1). Pearson’s correlation coefficient (*r*), Spearman’s correlation coefficient (ρ) and associated *p*-values are indicated. **b** Western blot showing expression of CD133 and KCNMA1 in ovc316 cultivated in standard media and media for induction of pluripotent stem cells for 8 days (*N* = 3). **c** Western blot showing expression of CD133 and KCNMA1 in cells treated with BMS-191011 and tetrandrine. Cells were treated for four days with 10 μM doses of KCNMA1 modulators (*N* = 3). **d** The density plot illustrating E-cadherin against CD133. Cells were treated for four days with 10 μM doses of KCNMA1 modulators BMS-191011 and tetrandrine and then analyzed by flow cytometer (*N* = 4). **e**, **f** The bar graphs show the effect of KCNMA1 modulation on the CD133 expression. Cells were treated for four days with 10 μM doses of KCNMA1 modulators BMS-191011, tetrandrine, Penitrem A, and then analyzed by flow cytometer (*N* = 4, DF 6 for Fig E; *N* = 3, DF 2 for Fig F). **g** The bar graphs represent median CD133 in cells treated with DMSO, BMS-191011 (10 μM), tetrandrine (5 μM), tetrandrine (5 μM)/DMSO, and tetrandrine (5 μM)/BMS-191011 (10 μM) daily for four days, and then analyzed by flow cytometer (*N* = 3, DF 8). **h** The XY plot and box plot visualizing separate and combined change in CD133 and E-cadherin relative to Mock. E-cadherin and CD133 changes were calculated by subtracting the replicate-matched Mock mean from the treatment mean for each marker after log1p transformation. The combined change was calculated as the Euclidean distance between these differences, representing the size of the change in both markers. The cells were treated with DMSO, BMS-191011 (10 μM), tetrandrine (5 μM), tetrandrine (5 μM)/DMSO, and tetrandrine (5 μM)/BMS-191011 (10 μM) daily for four days, and then analyzed by flow cytometer (*N* = 3, DF 6). Dots = replicates, white-centered dots = replicate-weighted mean. AF488 Alexa fluor 488, kDa Kilodalton, T/B Tetrandrine/BMS-191011.

Based on transcriptomic analysis of the TCGA [37], published studies corelating KCNMA1 expression levels with tumor progression [36, 38–41], and the recognized multifaceted role of calcium in cell (patho)physiology [42], we hypothesized that alterations of this ion channel could contribute to tumorigenesis as a regulator of cancer cell plasticity. Indeed, a positive correlation between expression of the *KCNMA1* and the *PROM1* gene (encoding the CD133 stem cell marker) was reported in some human tumors [9, 34], and this association was confirmed by our own analysis of the TCGA ovarian cancer dataset (Fig. 1a; [37]). Encouraged by such information from the literature and database mining, we next investigated experimentally whether *KCNMA1* expression is associated with ovarian cancer cell plasticity, focusing on two commonly used markers: the CD133 and E-cadherin [5]. First, in the human ovc316 ovarian cancer cell line grown in standard medium and in Nutristem medium that promotes the undifferentiated cellular state, the KCNMA1 abundance was higher when cells were cultured in the latter, and the trend in expression levels of KCNMA1 (detected by western blotting) followed that of the CD133 stemness marker (Fig. 1b, and Supplementary Data 1), suggesting a potential link between KCNMA1 and cancer cell plasticity in this model.

Next, to modulate the function of the KCNMA1 channel in standard media, we used the pharmacological modulators BMS-191011 and tetrandrine as channel activator and blocker, respectively [43, 44]. Channel activation consistently resulted in moderately lowered levels of CD133 by western blotting, which was, however, not reflected by flow cytometry, possibly because of KCNMA1’s epitope localization. Importantly, following channel blocking, cellular CD133 was similarly elevated in both assays (Fig. 1c–e, and Supplementary Data 1). Consistently, an upregulation of CD133 was also observed upon exposure of ovc316 cells to penitrem A, another pharmacological inhibitor of KCNMA1 (Fig. 1f) [45], indicating that the phenotypic response of this ovarian cancer model was not restricted to tetrandrine alone. Moreover, channel blocking resulted in a borderline significant increase of median E-cadherin protein levels (Fig. 1d, and Supplementary Fig. 1).

In separate 4-day time-course experiments involving repeated daily applications of the tetrandrine channel blocker on the cultured ovc316 cells, CD133 was upregulated (Fig. 1g, and Supplementary Fig. 2a), and shifted the culture to an E-cadherin-intermediate phenotype, thereby decreasing both the E-cadherin high and low fractions, while median E-cadherin levels of the culture were largely unaltered (Supplementary Fig. 2a,b). Notably, such repeated tetrandrine treatment led to a consistent increase specifically in the combined levels of E-cadherin and CD133 compared with mock-treated controls (Fig. 1h, and Supplementary Fig. 2a). This effect was further pronounced when tetrandrine-mediated channel inhibition was applied in a cyclical manner and alternated with BMS 191011-mediated channel activation, resulting in the maximal elevation of the combined E-cadherin/CD133 marker profile (Fig. 1h). These results suggest that the CD133 increase is associated with and may facilitate the more epithelial phenotype of less differentiated ovarian cancer cells.

To extend and validate the functional analysis of the KCNMA1 channel on additional human ovarian cell models, we then evaluated four other cell lines, three cancer-derived: Skov-3, UWB1.289, and OVCAR4, and one as a non-transformed counterpart: HOEC hTERT (Human Ovarian Surface Epithelial Cells). Using western blotting, we found that the Skov-3 and UWB1.289 cells displayed modest abundance of the KCNMA1 channel protein, while OVCAR4 and HOEC hTERT cells had no detectable KCNMA1 (Supplementary Fig. 3a, and Supplementary Data 2).

Next, consistently with the above functional analyzes for the ovc316 cells, the KCNMA1-expressing Skov-3 and UWB.289 cells responded by a phenotypic shift towards a CD133-high cell population after treatment with the channel blocker (tetrandrine), and showed only modest changes of CD133 expression when treated by the BMS-191011 channel activator, as shown by flow cytometry and western blotting (Supplementary Fig. 3b–d, and Supplementary Data 2). In contrast, the KCNMA1-negative control HOEC cells showed no detectable changes in CD133 expression in response to KCNMA1 modulation, while OVCAR4 cells had decreased, rather than elevated CD133 in response to tetrandrine (Supplementary Fig. 3e, f, and Supplementary Data 2). Skov-3 cells did not display any E-cadherin change upon modulation of the channel activity, while the UWB1.289 cells showed modestly increased and decreased E-cadherin after BMS-191011 and tetrandrine treatment, respectively (Supplementary Fig. 3b, c).

Overall, these findings indicate an impact of the KCNMA1 channel activity on plasticity of human ovarian cancer cell lines, suggesting KCNMA1’s potential role in the regulation of cellular (de)differentiation. Our data revealed reproducible correlations between the KCNMA1 states (protein presence and channel activity) and the CD133 stem cell marker. Channel blocking consistently led to CD133 upregulation, as shown by western blotting and flow cytometry. E-cadherin expression response was more variable among the different cell lines, and it was stable or elevated upon channel blocking in the ovc316 cells, indicating partial epithelial reinforcement, specifically in the CD133 positive cell subpopulation. KCNMA1 activation, on the other hand, had modest, if any, effect on E-cadherin. Notably, under the combined, oscillating treatment with tetrandrine and BMS-191011, the extent of the change seen for CD133 and E-cadherin relative to mock-treated controls was increased, reaching upregulation that was more pronounced than in cells treated with tetrandrine alone, as detected by flow cytometry.

Taken together, these initial findings suggest that oscillating, flexible modulation of KCNMA1 channel activity promotes the strongest shift towards a CD133-positive/E-cadherin-positive ovarian cancer cell phenotype.

### KCNMA1 expression level alters cancer cell growth and degree of differentiation

**Fig. 2:**
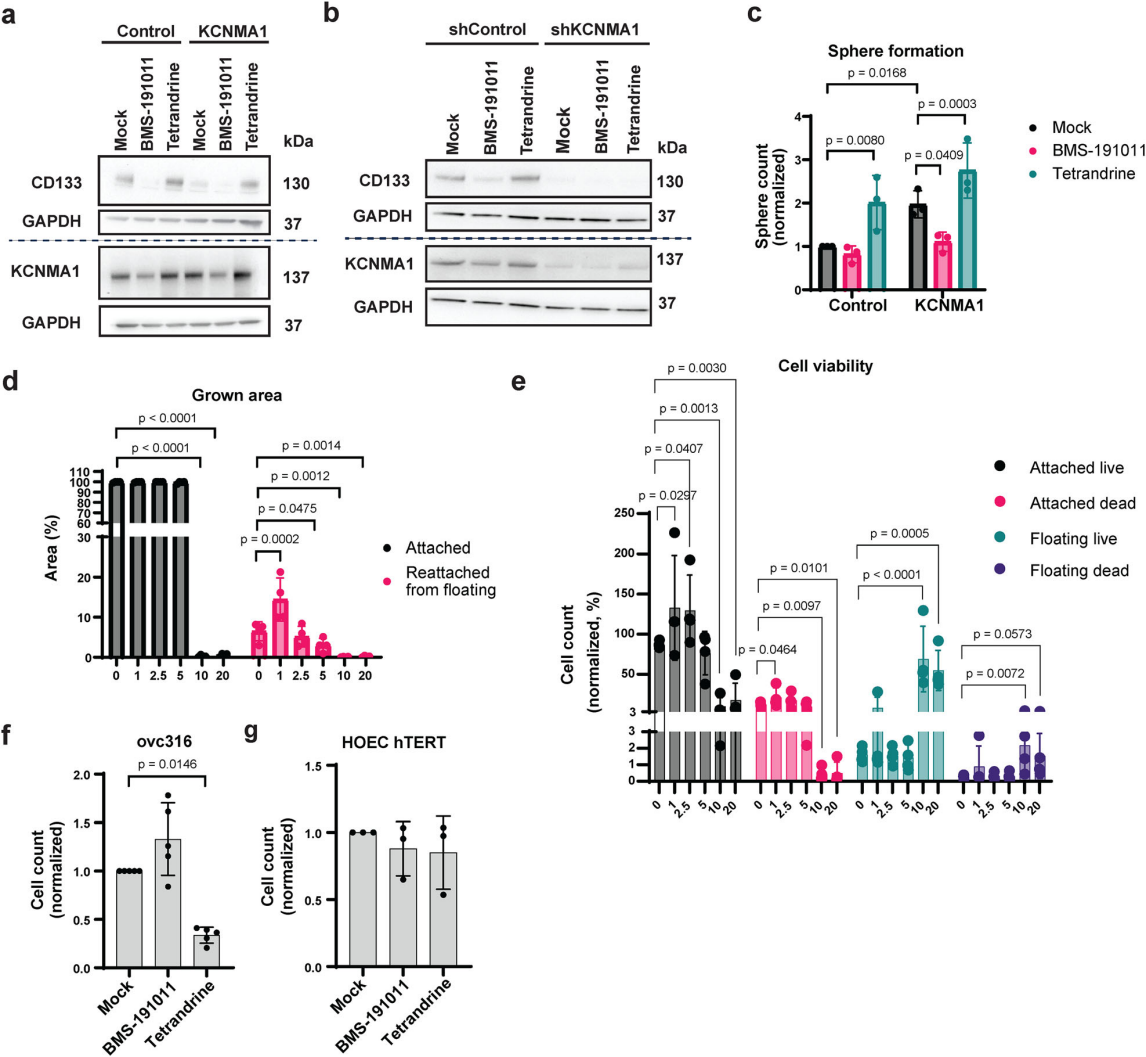
KCNMA1 expression and activity status regulate CD133, sphere formation potency and dissemination potential. **a** Western blot showing expression of CD133 and KCNMA1 in control and KCNMA1 overexpressing cells treated with BMS-191011 (10 μM) and tetrandrine (10 μM). Cells were treated for four days with 10 μM doses of KCNMA1 modulators (*N* = 2). **b** Western blot showing expression of CD133 and KCNMA1 in cells after control and KCNMA1 knockdown treated with BMS-191011 (10 μM) and tetrandrine (10 μM). Cells were treated for four days with 10 μM doses of KCNMA1 modulators (*N* = 3). **c** The bar graph of sphere formation assay comparing 3D growth in between cells overexpressing control and KCNMA1 vector. The cells were treated with low doses of channel opener (BMS-191011, 1 μM) and closer (tetrandrine, 1 μM) for 6 weeks. (*n* = 3, DF 10, *emmeans* used for marginal means estimation). **d** The bar graph depicting area of 12-well plate grown by cells. Floating cells were reseeded after four days of experiment to the new 12-well plate. After an additional four days, cell confluency (%) within the wells was evaluated. Cells were treated with a concentration gradient of tetrandrine for four days, followed by four days without treatment (*N* = 4, DF 15). **e** The bar graph depicting viability of cells after four-day treatment with increasing dose of tetrandrine and analyzed by flow cytometry (*N* = 4, DF 15). **f**, **g** Bar graph showing the results of a proliferation assay in ovc316 and HOEC hTERT cells. Cell numbers were normalized to the control grown. Cells were seeded under far-subconfluent conditions and treated with BMS-191011 (1 μM) or tetrandrine (1 μM) for 7 days (HOEC hTERT: *N* = 3, DF = 4, ovc316: *N* = 5, DF 8). E E-cadherin, kDa Kilodalton.

To complement the pharmacological manipulation of channel activity, we next varied expression levels of KCNMA1, using a lentiviral expression system in the syngeneic ovc316 cell line model (Supplementary Fig. 4a, and Supplementary Data 3). Overall, KCNMA1 overexpression was only tolerated at very modest levels, however it led to reduced total CD133 levels and increased expression of E-cadherin, as shown by western blotting and quantitative image-based microscopy (Fig. 2a, Supplementary Fig. 4b, Supplementary Table 1, and Supplementary Data 4). The KCNMA1-overexpressing cells were also analyzed in combination with KCNMA1 small-molecule modulators to examine any changes in CD133 and E-cadherin markers. KCNMA1-overexpressing cells responded to the channel modulators in a manner similar to control parental ovc316 cells with endogenous KCNMA1; treatment with BMS-191011 decreased, while tetrandrine increased CD133 (Fig. 2a, and Supplementary Data 4). Changes in E-cadherin levels were observed when comparing KCNMA1-overexpressing cells treated with either tetrandrine or BMS-191011, which led to increased or decreased E-cadherin, respectively (Supplementary Fig. 4b, and Supplementary Table 1).

As a complementary approach to overexpression, we next employed shRNA-mediated knockdown of KCNMA1 [46], resulting in an almost complete depletion of CD133, further supporting a functional role for KCNMA1 in maintaining cellular plasticity-associated traits. (Fig. 2b, and Supplementary Data 4).

Next, we examined the impact of KCNMA1 expression levels and its functional status upon pharmacological manipulation on the growth properties of the ovarian cancer cells in diverse complementary cell proliferation tests. The overall patterns of cell growth observed in: (i) sphere formation (3-D growth), (ii) wound healing/scratch assay, and (iii) very low-density cell seeding conditions, are presented in Supplementary Table 2, and the results of the individual assays are described in detail below.

Given the lower total CD133 protein pool among the KCNMA1-overexpressing cells compared to controls, we performed a sphere formation assay, comparing untreated cells with those treated with low doses of the channel activator and blocker, respectively. The otherwise untreated KCNMA1-overexpressing cells exhibited enhanced sphere formation compared to control cells, and this effect on sphere formation was suppressed and further potentiated by exposure of the cells to the channel activator and blocker, respectively (Fig. 2c, Supplementary Fig. 5a, and Supplementary Table 2).

Interestingly, in another cell growth-related, wound healing/scratch assay, the KCNMA1 overexpression was accompanied by slower regrowth of the cell culture surface area from which cells were scraped off in a cell monolayer, when compared to the control parental cells (Supplementary Fig. 5b, and Supplementary Table 2). Treatment with channel modulators revealed an opposing pattern: BMS-191011 enhanced such wound healing in control cells with endogenous KCNMA1 expression, whereas tetrandrine moderately promoted regrowth in the KCNMA1-overexpressing cells (Supplementary Fig. 5b). These differential responses are consistent with the notion that the biological, cell growth outcome is shaped by the basal cellular state, that is characterized for example by E-cadherin (see Supplementary Fig. 4b), a key epithelial adhesion molecule whose expression and localization reflect cell–cell contact integrity and epithelial differentiation [47].

Our findings so far have shown that altered KCNMA1 expression influenced cell growth. To further explore whether specific conditions could lead to changes in the cell growth patterns under channel manipulation, we next treated the ovc316 cells with increasing doses of the channel inhibitor tetrandrine. We observed concentration-dependent detachment of the cells from the surface (Fig. 2d). After 4 days of treatment, floating cells detached from the cell culture surface were collected, washed and re-seeded without tetrandrine for another 4 days, and live versus dead cell counts were estimated at the cell re-seeding (Fig. 2e).

Notably, after re-seeding in the absence of the channel blocker, the cells were able to re-attach to the surface after the original 4-day treatment with tetrandrine concentrations of up to 5 µM, with the most efficient cell re-attachment observed after exposure to 1 µM tetrandrine (Fig. 2d). While tetrandrine-treated cells exhibited slightly higher cell death compared to mock-treated cells, mainly from the 10 µM drug concentration, this result may be obscured by the disintegration of some floating cells after death (Fig. 2e).

Since we observed that low-dose tetrandrine potentiated the 3-D spheroid growth but also induced cell detachment, we next performed a cell proliferation assay in which the ovc316 and control HOEC hTERT cells were seeded at low cell density and treated with low doses of either channel modulator (Fig. 2f, g, and Supplementary Table 2). The ovc316 cells exhibited a moderate growth advantage under treatment with BMS-191011, whereas exposure to the channel blocker tetrandrine strongly inhibited their low-density growth. No such effects on cell growth under low-density 2-D conditions were observed in the non-transformed control HOEC hTERT cells.

These results suggest a potential clinical relevance of KCNMA1 abundance and activity. Cells overexpressing the KCNMA1 channel exhibited enhanced growth under 3-D cell culture conditions but showed reduced repopulating capacity in the scratch assay. Additionally, we uncovered a potential role of channel modulation in metastasis-associated cell traits, as cells treated with the channel blocker became detached from the cell culture surface and floated, while at least partly retaining viability. Furthermore, floating cells were able to re-attach once the treatment was discontinued, indicating the importance of dynamic channel activity regulation. The results of our low cell density adherent cell proliferation assays suggested that channel blocking by tetrandrine effectively attenuated cell proliferation in this setting. On the other hand, low-dose tetrandrine exposure potentiated the 3-D spheroid cell growth, increased repopulation capacity of the KCNMA1 overexpressing cells in the wound healing scratch assay, and induced cell detachment, leading to altered cellular states and biological behavior. In contrast, activation of the channel by BMS-191011 treatment had the opposite effect, as this enhanced the cancer cell proliferation under adherent, low-density cell culture conditions, increased repopulation of control cells with endogenous levels of KCNMA1 in the wound healing/scratch assay, and attenuated the 3-D spheroid growth. Finally, we suggest the importance of the basal cellular state as decisive for cell behavior in response to KCNMA1 modulation.

### Distinct calcium–potassium signatures define epithelial and stem-like cancer cell states

**Fig. 3:**
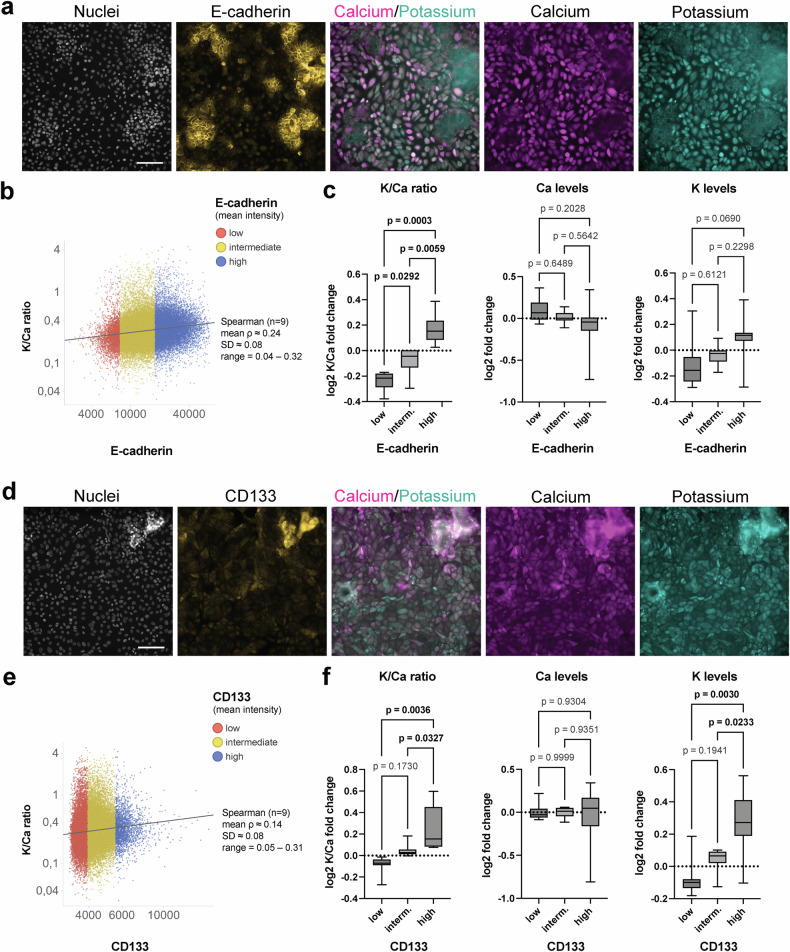
Single-cell stratification by E-cadherin and CD133 reveals distinct calcium–potassium signatures. **a** Representative quantitative image-based cytometry (QIBC) images of low-passage ovc316 cultures stained for nuclei, E-cadherin, cytosolic calcium and potassium (single channels and overlay). Scale bar, 100 µm. **b**, **c** Scatter plots and bar graphs showing stratification of single-cell calcium, potassium and potassium/calcium (K/Ca) ratio according to E-cadherin abundance. Increasing E-cadherin levels associate with progressive elevation of the K/Ca ratio, while absolute calcium and potassium levels remain comparable between gates. Single-cell scatter analysis indicates a positive correlation between calcium and potassium signals. **d** Representative QIBC images of low-passage ovc316 cultures stained for nuclei, CD133, cytosolic calcium and potassium (single channels and overlay). Scale bar, 100 µm. **e**, **f** Scatter plots and bar graphs showing stratification of single-cell calcium, potassium and K/Ca ratio according to CD133 abundance. High CD133 levels associate with increased intracellular potassium and broader calcium distributions. Calcium–potassium correlation is reduced compared to E-cadherin stratification. Data represent paired analyzes of independent experiments (*N* = 9, DF = 6, *emmeans*). Acquisition of ion dyes (live cells) was aligned (XY shift) with acquisition of antibodies (fixed cells) to correlate for analysis. For each experiment, single-cell measurements were stratified into the indicated gates (color; low, intermediate, high) and summarized as log2 fold changes of gate-specific median fluorescence relative to the corresponding culture median following within-experiment normalisation. Interm. Intermediate.

To examine how intracellular ionic states relate to phenotypic heterogeneity, we quantified cytosolic calcium (Oregon Green 488 BAPTA-1 AM) and potassium (PBFI AM) in low passage ovc316 cells at single-cell resolution by QIBC and stratified these measurements independently according to E-cadherin or CD133 abundance (Fig. 3). Stratification by E-cadherin revealed a pronounced progressive increase in the potassium/calcium (K/Ca) ratio consistent with increasing E-cadherin positivity (Fig. 3b, c). These changes were accompanied by relatively coordinated variation of calcium and potassium levels that did not significantly differ in absolute values individually (Fig. 3c).

In contrast, CD133-high cells displayed a distinct ionic signature. While also associated with elevated K/Ca ratio (Fig. 3e, f), these cells retained the highest cytosolic potassium levels and exhibited markedly broader calcium distributions, reflected by a ~3–5-fold increase in inter-culture calcium variability compared with CD133-low populations (Supplementary Table 1). Although CD133-high cells remained positively associated with elevated K/Ca ratios, the calcium–potassium relationship was less constrained than across the epithelial range (Fig. 3b, e), consistent with greater heterogeneity in calcium levels, while potassium retention was preserved. Together, these data identify a potassium-enriched and calcium-variable ionic state associated with stem-like CD133-positive cancer cells that is related to, but distinct from, the ionic patterns observed across the epithelial–mesenchymal range.

### KCNMA1 regulates cancer cell plasticity through calcium–potassium ion balance

**Fig. 4:**
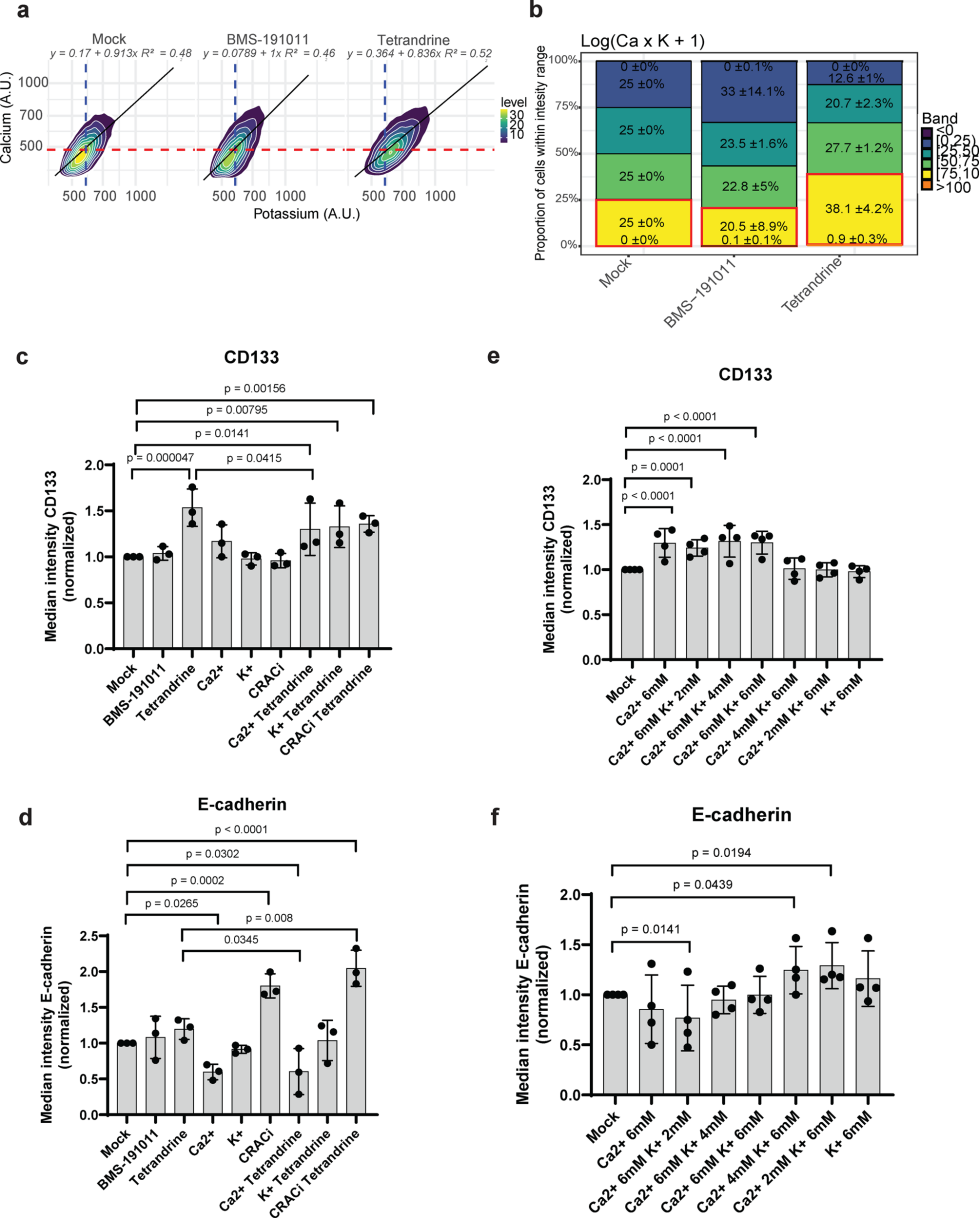
KCNMA1 closing balances the calcium/potassium ratio to stabilize the E/M hybrid state under calcium-driven EMT. **a** Density scatter plot of ScanR microscopy-based cytometry of live cells showing cytoplasmic median intensity of calcium and potassium stained with dyes. Cells were treated with BMS-191011 (5 μM) and tetrandrine (1 μM) for four days (*N* = 3). Regression equations for all replicates were as follows: Mock: *y* = 0.17 + 0.913*x* (*R*² = 0.48), *y* = 0.381 + 0.834*x* (*R*² = 0.41), *y* = 0.29 + 0.853*x* (*R*² = 0.52); BMS-191011: *y* = 0.0789 + 1*x* (*R*² = 0.46), *y* = 0.228 + 0.889*x* (*R*² = 0.40), *y* = 0.396 + 0.816*x* (*R*² = 0.52); Tetrandrine: *y* = 0.364 + 0.836*x* (*R*² = 0.52), *y* = 0.679 + 0.72*x* (*R*² = 0.38), *y* = 0.466 + 0.786*x* (*R*² = 0.49). **b** 100% stacked bar graph comparing log-transformed integrated calcium/potassium metric calculated by multiplying calcium and potassium intensities on single-cell level. Plot shows differences between treatments relative to Mock. The bars contain proportion of cells ± SD in subpopulation (significant bars are with red stroke). Ovc316 cells were treated with BMS-191011 (5 μM) or tetrandrine (1 μM) for four days (*N* = 3, *emmeans*, DF = 4). **c**, **d** The bar graphs depicting the of various calcium modulators with or without tetrandrine cotreatment effect, on CD133 and E-cadherin expression. Cells were treated for four days with BMS-191011 (10 μM), tetrandrine (10 μM), calcium (5 mM), potassium (5 mM), CRACi (1 μM), or by combinations of tetrandrine with listed chemicals, and then analyzed by flow cytometer (*N* = 3, DF = 16 overall; DF = 6 for comparisons involving tetrandrine-treated groups only). **e**, **f** The bar graphs depicting the effect of various calcium and potassium ratios on the CD133 and E-cadherin expression. Cells were treated for four days with different ratios of calcium and potassium and then analyzed by flow cytometer (*N* = 4, DF = 21). A.U. Arbitrary Unit.

Having identified distinct calcium–potassium signatures associated with epithelial and stem-like states, we next examined whether direct modulation of KCNMA1 activity reshapes these intracellular ionic parameters. We treated ovc316 cells with either BMS-191011 or tetrandrine for 4 days, followed by measurements of potassium and calcium levels. We observed differentially altered calcium and potassium content following BMS-191011 and tetrandrine treatment, respectively (Fig. 4a, b, Supplementary Fig. 6a, b, and Supplementary Table 1). Linear regression analysis of cytosolic calcium and potassium showed altered differences in slope. While BMS-191011 increased the slope to 0.9 ± 0.09 compared to mock (0.87 ± 0.04), indicating an enhanced ratio of calcium to potassium, tetrandrine decreased the slope to 0.78 ± 0.06, revealing an attenuated calcium increase in relation to potassium. The *R*² values ranged from 0.38 to 0.52, suggesting a moderate positive relationship between calcium and potassium (Fig. 4a). Despite the observed changes in the relationship between calcium and potassium on the single cell level, the integrated metric (Ca x K) showed no overall significant response to BMS-191011 but a combined increase after tetrandrine treatment (Fig. 3b, and Supplementary Table 1). Further analysis of the data uncovered that potassium levels were generally increased by tetrandrine (Supplementary Fig. 7a, b, and Supplementary Table 1).

Calcium and potassium play complex roles in physiology, regulating numerous cellular processes, including membrane polarization [48, 49]. Importantly, the subcellular distribution of KCNMA1 is diverse and not restricted to cell membranes only. KCNMA1 has also been reported to localize to nucleus, Golgi, endoplasmic reticulum, as well mitochondria [50], a distribution that we confirmed by immunocytochemistry in ovc316 and control normal retinal pigmented epithelial (RPE-1) cells (Supplementary Fig. 7). As a readout for metabolic parameters related to calcium-potassium homeostasis in context with KCNMA1, we measured mitochondrial membrane potential (ΔΨm) using MitoTracker Deep Red FM, a dye that accumulates in active mitochondria as a consequence of their polarized membranes. When fractionated for different degrees of positivity, we observed that the subpopulation of ΔΨm high cells (75–100%) increased following pharmacological channel blocking, whereas channel activation had no effect, indicating rather active KCNMA1 channels as a ground state in ovc316 cells (Supplementary Fig. 6c, and Supplementary Table 1).

We further investigated the phenotypic impact of calcium and potassium on ovc316 cells more directly by altering levels of calcium or potassium, and by blocking calcium influx using the calcium release-activated channel (CRAC) inhibitor YM-58483 (Fig. 4c, d, and Supplementary Fig. 8a–c). The addition of calcium to the culture medium led to a mild increase in the fraction of CD133-positive ovc316 cells, with or without tetrandrine co-treatment, particularly within the CD44-high/E-cadherin-high subpopulation, marking the E/M hybrid state in ovarian cancer cultures [4, 5]. This shift was further accompanied by an overall decreased E-cadherin expression, indicating the induction of EMT (Fig. 4d). Potassium and the CRAC inhibitor were each capable of interfering with tetrandrine activity, while the CRAC inhibitor strongly induced E-cadherin expression (Fig. 4c, d, and Supplementary Fig. 8a–c).

Given that KCNMA1 functions as a calcium-activated potassium channel, we further confirmed the mutual regulation between calcium and potassium. To explore this relationship, we treated cells with different calcium/potassium ratios. Again, high calcium levels (6 mM) consistently elevated CD133, despite increasing amounts of potassium. In contrast, high levels of potassium (6 mM) counteracted the loss of E-cadherin, and further decreasing the calcium/potassium ratio opposed CD133 induction and moderately raised E-cadherin levels (Fig. 4e, f, and Supplementary Fig. 8d).

Our observations suggest a mechanistic basis for KCNMA1-mediated modulation of cellular plasticity. KCNMA1 activity alters cytosolic potassium and calcium levels. Cells treated by BMS-191011 and tetrandrine exhibited lower and higher calcium/potassium content than mock-treated cells, respectively, suggesting a dependence of CD133 expression on calcium and potassium. Furthermore, we showed strong functional coupling between calcium and potassium, and this coupling was strengthened and weakened by BMS-191011 and tetrandrine treatments, respectively, suggesting the importance of the absolute concentration of these ions but also their ratio to each other. In addition to functional coupling, we validated the opposing effect these ions have on CD133 and E-cadherin expression. Altogether, these findings uncover a regulatory interplay between calcium and potassium. The balance between their coupling and natural feedback regulation might be the key underlying the different responses driven by basal cellular states, ultimately leading to a decision on how cells respond to channel modulation. We were also able to manipulate the CD133-high cell population through direct calcium, potassium, and CRAC channel inhibitor treatment. We further verified the importance of the basal cellular state by analysis of cell subpopulations (CD44 high/E-cadherin high), uncovering effects otherwise hidden by the bulk cell population.

### KCNMA1 manipulation alters cancer cell sensitivity to chemotherapy drugs

**Fig. 5:**
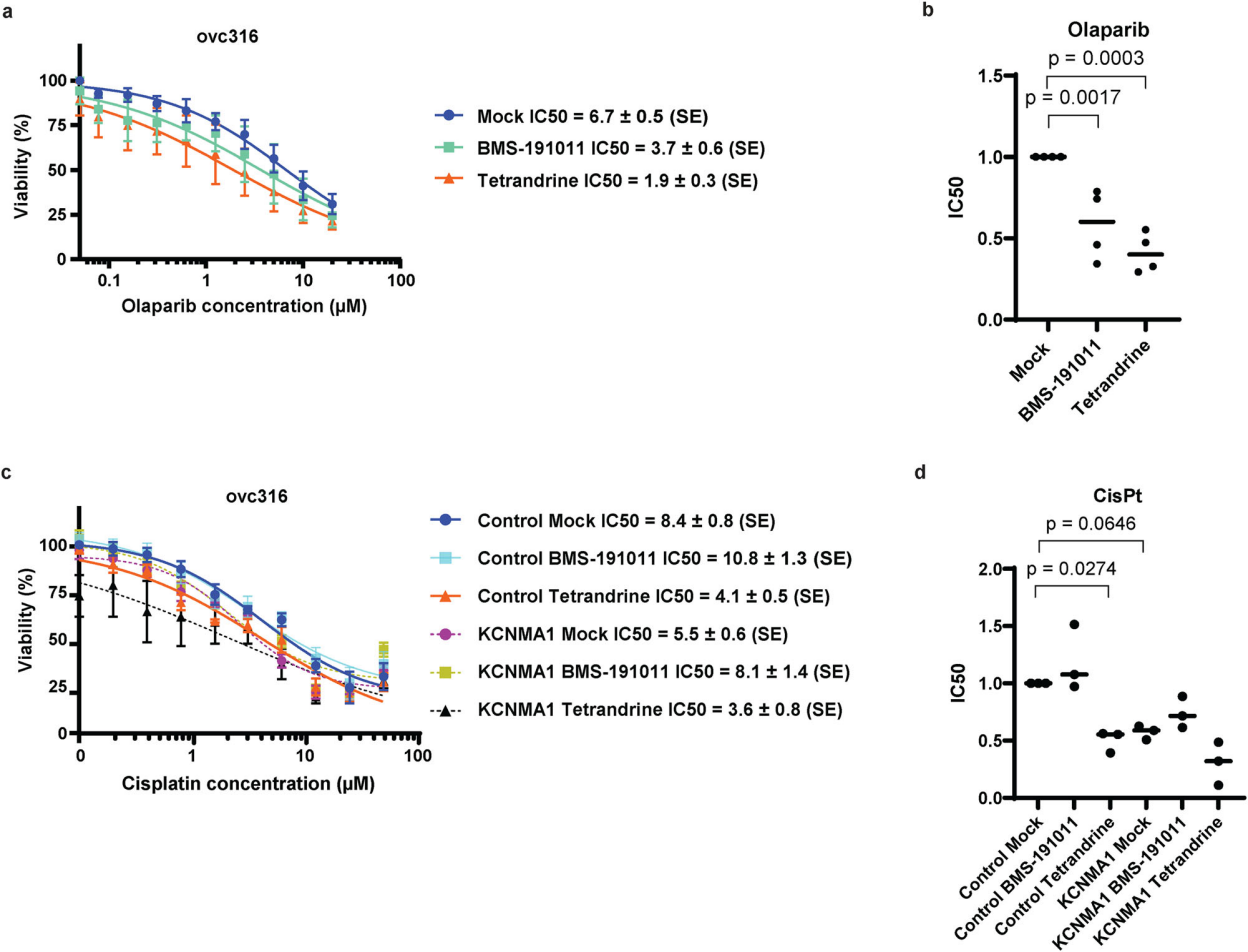
KCNMA1 modulation alters cellular sensitivity to the standard-of-care chemotherapy in ovarian cancer cells. **a**, **c** XY line graphs showing the cytotoxicity of increasing Olaparib (in ovc316) and cisplatin (in control and KCNMA1 overexpressing ovc316) concentrations co-treated with BMS-191011 (1 µM) and tetrandrine (1 µM). **b**, **d** The bar graph comparing IC50s derived from data sets plotted in A and C. Olaparib: statistics of normalized data, *N* = 4, DF = 6; cisplatin: statistics of normalized data, *N* = 3, DF = 10 (*emmeans*). NT non-treated, SE standard error.

Notably, tetrandrine as well as BMS-191011 have been used in clinical trials and preclinical treatment studies, respectively [51, 52]. Here, given our present findings on the impact of the calcium-activated potassium channel activity modulation on cancer cell plasticity and growth patterns, we also performed cell cytotoxicity assays, first combining the KCNMA1 modulation with increasing doses of Olaparib, a PARP inhibitor clinically approved for the treatment of ovarian carcinomas [53]. Specifically, tetrandrine effectively enhanced the tumor cell toxicity of Olaparib, as reflected in the lower IC50 for Olaparib when combined with the channel inhibitor (Fig. 5a, b). Notably, the BMS-191011 channel activator also increased Olaparib cytotoxicity (Fig. 5a, b).

Furthermore, to examine whether the observed chemotherapy-potentiating effects of BMS-191011 and tetrandrine were applicable more broadly, we performed cytotoxicity assays also in combination with cisplatin, an old chemotherapeutic drug widely employed in oncology, including in the treatment of ovarian cancer [54]. In this experiment, we also included the KCNMA1 overexpressing ovc316-derived cell line to assess any potential effect of the overabundant channel protein. Overall, both ovc316 cell lines exhibited increased sensitivity to cisplatin treatment when combined with the channel blocker tetrandrine (Fig. 5c, d). In contrast, and unlike in the combination with Olaparib, the BMS-191011 channel activator showed no additional cytotoxic effect when combined with cisplatin treatment in both ovc316 cell lines.

Taken together, the cell toxicity assays suggest that tetrandrine has the potential to enhance the efficacy of standard chemotherapy. Interestingly, while BMS-191011 increased the cytotoxicity of Olaparib, it had no synergistic effect with cisplatin toxicity, implying drug-dependent differential effects in our ovarian cancer cell model.

To explore the broader relevance of ion channel dysregulation in ovarian cancer, we investigated publicly available genomic datasets [55, 56] using the cBioPortal platform [57–59] (Supplementary Fig. 9). KCNMA1 itself was altered in ~6% of cases, while related ion channel genes were altered in ~53% of tumors, predominantly through copy-number gains. These findings indicate that calcium-activated potassium signaling is frequently perturbed in ovarian cancer.

## Discussion

Our study shows that maintaining a balanced ratio between calcium and potassium buffers transitions between epithelial and mesenchymal phenotypes and thereby preserves the E/M hybrid state as well as cellular stemness in ovarian cancer cells. We identify the calcium-activated potassium channel KCNMA1 as a key regulator of this buffering mechanism, which enables cells to counteract environmental cues that induce phenotypic changes. This ion control mechanism links KCNMA1 activity to cancer cell identity, adaptability, and therapeutic response, and suggests that ion channel regulation could be utilized to improve therapeutic outcomes in ovarian carcinoma.

Using transcriptomic analysis of the TCGA ovarian cancer dataset, we found that KCNMA1 expression positively correlates with the *PROM1* gene encoding CD133 in ovarian cancer, in line with previous reports suggesting a role for KCNMA1 in promoting disease progression in other malignancies [26, 31]. Consistent with this finding, KCNMA1 and CD133 were both amplified when cultured ovarian cancer cells were maintained in media promoting the undifferentiated state. Functional assays demonstrated that pharmacological inhibition of KCNMA1 with low-dose tetrandrine induced CD133 upregulation and shifted cultures toward an E-cadherin–intermediate state or, depending on the initial cellular background, even increased surface E-cadherin. In contrast, KCNMA1 activation with BMS-191011 reduced CD133 levels. These data suggest that KCNMA1 regulates epithelial plasticity via coordinated calcium–potassium signaling rather than through static control of individual stemness-associated traits. Induction of channel oscillation by alternating treatment with BMS-191011 and tetrandrine further enhanced the combined CD133 and E-cadherin phenotype, supporting the idea that maintenance of the E/M hybrid state relies on regulated calcium–potassium balancing, a new insight that extends the currently limited knowledge about the role of KCNMA1 in cancer biology in general [60–62], and ovarian carcinomas in particular [63]. Because cytosolic calcium signaling occurs as transient spikes driven by circadian, metabolic and microenvironmental cues [64, 65], these fluctuations are expected to trigger periodic KCNMA1 opening and controlled potassium efflux, followed by channel closure as calcium levels decline. In line with this model, CD133-high cells showed broad variation in total calcium but overall high potassium content at the single-cell level. Thus, plastic tumor cells appear to be defined by stabilised ionic balance during calcium fluctuations rather than uniformly increased calcium signaling. Oscillatory channel modulation may therefore model physiologically relevant ionic stress cycles shaping epithelial–mesenchymal plasticity and therapy tolerance.

At the cellular level, pharmacological modulation of KCNMA1 reshaped cytosolic calcium–potassium flux and thereby influenced phenotypic transitions. Channel inhibition increased intracellular potassium and elevated the combined calcium–potassium content, consistent with a known tetrandrine-induced redistribution of intracellular calcium stores [44]. Supplementing cells with calcium led to an induction of CD133 and concomitant loss of E-cadherin, indicating EMT, while gradually elevating potassium moderately raised E-cadherin levels. Tetrandrine increased both ions in a correlative manner, but also shifted the balance toward potassium, whereas BMS-191011 favored calcium dominance over potassium at the single-cell level.

These findings also explain our earlier observation that ovarian tumors and xenografts derived thereof are enriched in epithelial and E/M hybrid cells. Of these, only E/M hybrid cells enriched in cancer stem-like cells can adapt to long-term in vitro culture, where they then undergo EMT and rapidly lose the stemness phenotype upon passaging. Furthermore, only cultures containing E/M hybrid cells were able to form tumors, while sub-clonal cultures restricted to either epithelial or mesenchymal phenotypes had no tumor-forming ability [5]. While previous reports have already suggested calcium-activated potassium channels as contributors to stemness and EMT [26, 61], these studies did, however, not address how epithelial cells preserve their identity while activating EMT-associated pathways.

**Fig. 6:**
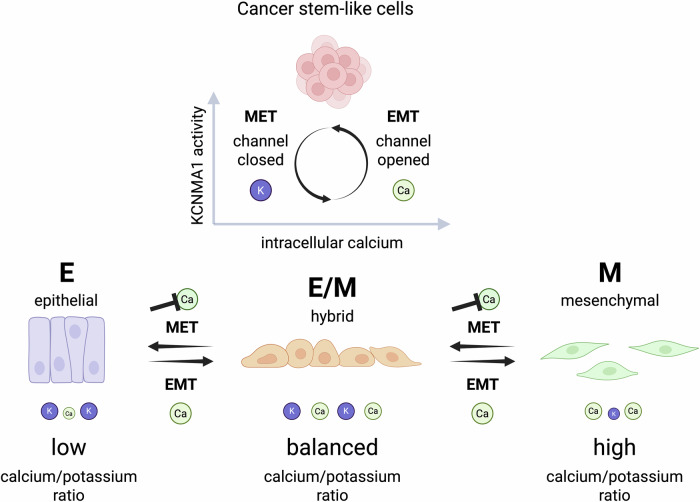
KCNMA1-mediated calcium-potassium balancing maintains the epithelial–mesenchymal hybrid state in ovarian cancer. Model illustrating how the calcium-activated potassium channel KCNMA1 regulates cytosolic Ca and K levels to control epithelial–mesenchymal plasticity. Under balanced Ca/K conditions, KCNMA1 activity buffers calcium-driven EMT signaling while stabilizing epithelial features such as adherens junctions. This balance enables maintenance of the epithelial/mesenchymal (E/M) hybrid state, which is closely linked to stemness, adaptability, and tumor-initiating capacity. Partial channel inhibition (e.g., low-dose tetrandrine) leads to cytosolic K accumulation, supporting epithelial integrity while allowing episodic Ca increases to activate EMT-linked stemness cues, together reinforcing the hybrid state. In contrast, channel activation or unbuffered Ca influx shifts the Ca/K ratio toward calcium dominance, promoting EMT and loss of epithelial identity. Conversely, strong K dominance favors mesenchymal-to-epithelial reversion (MET) and epithelial stabilization, when cytosolic calcium levels decline. EMT epithelial–mesenchymal transition, MET mesenchymal–epithelial transition, K potassium, Ca calcium. Created in BioRender. Strauss, R. (2026) https://BioRender.com/xknregu.

Our data support a model in which KCNMA1-mediated dynamic calcium–potassium balancing buffers calcium-driven EMT signaling rather than preventing it. Cancer stem-like cells preferentially retain KCNMA1 expression and sustained intracellular potassium, which helps preserve epithelial integrity, whereas transient calcium elevations promote EMT induction. By limiting prolonged calcium dominance, KCNMA1-dependent ion balancing stabilizes the E/M hybrid state by permitting repeated transient EMT engagement that promotes stemness enrichment while preventing full mesenchymal conversion (Fig. 6).

We propose that such ionic balancing also underlies the context-dependent effects of KCNMA1 reported in the literature. While KCNMA1 promotes invasion and proliferation in glioblastoma and breast cancer, it has tumor-suppressive roles in gastric cancer [34–36, 66]. Our data suggest a mechanistic explanation: in 2-D adherent cultures, prone to undergo EMT, prolonged channel inhibition induced cell detachment and reduced proliferation, while it promoted the E/M hybrid state and fueled 3-D spheroid cultures with epithelial characteristics. The enhanced dissemination and sphere-forming potential observed after tetrandrine treatment is therefore also in line with the previously reported enhanced metastatic potential of E/M hybrid cells during the very early stages of EMT [67]. In contrast, extended channel activation in our hands enhanced wound healing and proliferation in 2-D, while 3-D growth was inhibited. The context-dependent action of KCNMA1 therefore leads to different outcomes based on environmental cues that trigger either EMT or MET due to calcium or potassium dominance, respectively.

Finally, our study demonstrates that prolonged KCNMA1 modulation in either direction, activation or blockage, sensitizes ovarian cancer cells to currently used chemotherapy drugs. The response to cisplatin was enhanced by tetrandrine, confirming earlier studies [68–70], while sensitivity to Olaparib, which has not been previously assessed, could be increased by both tetrandrine and BMS-191011. KCNMA1 blockage also increased mitochondrial polarization, consistent with altered bioenergetics in plastic subpopulations. The enhanced Olaparib response under both treatment conditions supports the idea that PARP inhibitor resistance may be particularly pronounced in plastic E/M hybrid cells that rely on calcium-potassium balancing to sustain mitochondrial and also proliferative function in addition to their transitional phenotype [5, 71, 72]. Although a range of KCNMA1 blockers have shown complications, tetrandrine, as well as channel activators such as BMS-191011, have been well tolerated when tested in clinical or pre-clinical settings [51, 52], indicating their potential use in targeted cancer therapy. While our study mainly focuses on the mechanistic framework of calcium-potassium balancing in ovarian cancer cells, future research should assess the in vivo efficacy of KCNMA1 modulation in patient-derived xenografts or organoid models and explore downstream regulators such as YAP/TAZ and NFAT that may link ion homeostasis to transcriptional control of plasticity [73–76]. Consistent with a broader role of ion homeostasis in ovarian cancer, analysis of publicly available genomic datasets showed that more than half of tumors harbor alterations in genes encoding calcium-activated potassium channels and related ion regulators (Supplementary Fig. 9). Although KCNMA1 itself is altered in a smaller subset of cases, recurrent changes in functionally related channel genes suggest that calcium–potassium balancing represents a commonly perturbed regulatory axis. This raises the possibility of functional compensation within this channel network and indicates that similar ion-balancing mechanisms may operate in KCNMA1-low tumors.

In conclusion, our study describes cytosolic calcium-potassium balancing as a key mechanism that buffers transitions between epithelial and mesenchymal phenotypes. This ion-balancing mechanism is modulated by KCNMA1, which preserves the stemness-associated E/M hybrid phenotype, metabolic state, as well as resistance to current treatment options. Interfering with its ion channel modulation to reduce cellular plasticity, therefore, represents a promising therapeutic approach in ovarian cancer, especially when combined with treatment targeting less plastic cancer cell states.

## Materials and methods

### Cell lines and cell culture media

Ovarian cancer cell lines (OVCAR4, Skov-3, UWB1.289 originating from ATCC, ovc316 [13], and human ovarian surface epithelial cells (HOEC, Innoprot) immortalized by hTERT were cultured in MEGM/DMEM media (PromoCell/Gibco, 1:1) supplemented with a vial of SupplementMix (PromoCell), 5% FBS (Gibco) and 0.5% Penicillin/Streptomycin (Gibco). Retinal pigment epithelial cell line immortalized with hTERT (hTERT RPE-1, ATCC) was cultivated in DMEM supplemented with 10% FBS and 0.5% Penicillin/Streptoimycin. For the induction of pluripotent stem cells, the ovc316 cells were cultured for 8 days in Nutristem hPSC XF culture media (Reprocell).

### Stable cell line establishment

Cells were seeded in a 24-well plate at confluency of 70–90%. The following day, an infection with the lentiviral construct was conducted. After another 24 h, the media was changed, and appropriate antibiotics (Blasticidin, Hygromycin) were added. Selection was carried out in parallel with control/noninfected cells. The following cell lines were established: ovc316 overexpressing control vector, KCNMA1 (VB900125-8204evy), and ovc316 cells with knockdown of shCon, shKCNMA1 (VB220125-1207emr, target sequence: CCCTGAAATCATAGAGTTAAT, reported 80% efficacy) [46].

### Flow cytometry

Cells were seeded in a 6-well plate and treated with chemicals as described in the figure legends. Cells were harvested using Versene (Gibco) or TrypLE Express (Gibco). After detaching, the cells were washed with complete DMEM media, spun down, and resuspended in 50 µl of 1× PBS containing 1% FBS and antibodies and dye: Anti-CD133/1 (AC133)-APC (Miltenyi Biotec, 130-113-106), Anti-CD44-PE (BD Bioscience, 555479), Anti-CD324-Alexa Fluor 488 (BioLegend, 324110), Hoechst 33258 (Invitrogen, H3569). Cells were incubated on ice for 30 min and then washed in 1× PBS containing 1% FBS. Samples were run on the Cytek Aurora (Cytek Biosciences) and analyzed using FlowJo (BD Biosciences/FlowJo LLC, version 10.9.0). An example of a gating strategy is shown in Supplementary Fig. 10.

### Western blotting

Equal amounts of protein lysates were separated on gradient Bis-Tris or Tris-Acetate Protein Gels (Thermo Scientific). The separated proteins were transferred onto a nitrocellulose membrane using the iBlot system (Invitrogen). The membrane was blocked with 1× PBS containing 0.1% Tween-20 and 5% powdered milk (BioRad) for 1 h at room temperature, then incubated overnight at 4 °C with primary antibodies and for 1 h at room temperature in secondary antibodies. Tagged antibodies were detected using ChemiDoc (BioRad) after the addition of substrate (Thermo Scientific, 32132). Following antibodies were used: Anti-Maxi K/BK (Merck, ZRB2180), Anti-CD133 (Cell Signaling, 64326S), GAPDH (GeneTex, GTX627408), Goat Anti-Rabbit IgG (H+L) Peroxidase (Vector Laboratories, PI-1000-1), Horse Anti-Mouse IgG (H+L) Peroxidase (Vector Laboratories, PI-2000).

### Quantitative image-based cytometry

Cells were seeded in a 96-well plate (Revvity, 6055300) and treated as described in the figure legends. Cytosolic potassium was measured by PBFI AM (a potassium-sensitive ratio metric fluorophore, hydrolyzing to PBFI with sodium-dependent potassium affinity, [77]) and calcium assessed by Oregon Green 488 BAPTA-1 AM (a fluorescent probe conjugated to a calcium chelator increasing fluorescence intensity free cytosolic calcium binding, without accumulation in organelles [78, 79]). Images for potassium and calcium measurements were acquired on live cells, and then matched with the same position after fixation to align with antibody staining using XY-shift adjustment. MitoTracker Deep Red FM was incubated on live cells and measured after fixation. Cells were fixed with 4% PFA and permeabilized with 0.5% Triton X-100 in 1× PBS. After permeabilization, the cells were incubated with selected antibodies, followed by staining with secondary antibodies and DAPI. The cells were mounted using Flouromount-G (SouthernBiotech/AH Diagnostics, 0100). For experiments in Fig. 3, live cell images were matched with fixed cells via XY-shift. Images were acquired using a high-throughput screening ScanR microscope (Evident) and analyzed with the linked ScanR Analysis software (Evident, version 3.4.1). RStudio, GraphPad Prism, or TIBCO Sportfire were used for visualization of scanR data.

Following antibody and/or dyes (used according to the manufacturer instructions) were used: MitoTrackerTM Deep Red FM (Invitrogen, M22426), OG 488 BAPTA-1 AM (Tocris, 6256), PBFI AM (Cayman chemicals, 21602), DAPI (Invitrogen, D1306), Anti-CD133 (Cell Signaling, 64326), Anti-E-cadherin (Cell Signaling, 3195; BD transduction laboratories, 610181). Goat anti-Rabbit IgG (H+L) Alexa Fluor 750 (Invitrogen, A-21039), Goat anti-Rabbit IgG (H+L) Alexa Fluor 647 (Invitrogen, A-21245), Goat anti-Mouse IgG (H+L) Alexa Fluor 488 (Invitrogen, A-11029).

### Tumor sphere formation assay

Cells were seeded in serum-free media in a low attachment plate (Corning, 3473) at a density of 30.000 cells per well. The cells were treated as described in the figure legends. After 6 weeks, sphere count and size were measured using a Celigo Image Cytometer (Revvity).

### Viability/reattachment assay

Cells were seeded in duplicate in a 12-well plate and treated with tetrandrine as described in the figure legends. Attached and floating cells from one well of the duplicate were harvested using TrypLE Express (Gibco). After detaching, the cells were washed with complete DMEM media, spun down, and resuspended in 50 µl of 1× PBS containing 1% FBS and Hoechst 33258 dye (Invitrogen, H3569). Cells were incubated on ice for 30 min and then washed in 1× PBS containing 1% FBS. Samples were run on the Cytek Aurora (Cytek Biosciences).

The second well of duplicates was used for further experiments evaluating the reattachment of floating cells. Floating cells were collected, washed with 1× PBS, spun down, and reseeded in fresh media without treatment in a new 12-well plate. Attached cells were washed with 1× PBS, and fresh media were added. The cells were cultured for an additional 4 days and then fixed in crystal violet. The area of wells covered by cells was measured using a Celigo Image Cytometer (Revvity).

### Cell cytotoxicity assay after chemotherapy exposure

Cells were seeded in triplicate in a 96-well plate. After 4 days, they were treated with a concentration gradient of Olaparib (Selleckchem, S1060) and cisplatin (NOMECO, 598049), using the Agilent Bravo SRT, as described in the figure legend. Five days post-treatment, cells were stained with Hoechst 33342 (Invitrogen, H3570) and propidium iodide (Invitrogen, P1304MP) for 15 min. Fluorescence was then analyzed using the Celigo Image Cytometer (Revvity).

### Cell motility scratch assay

Cells were seeded in 12-well plates and treated with 2.5 µM and 5 µM concentration of channel modulators. The scratch in the cell monolayer was made by 1 ml pipette tip after 4 days of cell growth, and the cell-free area of the scratch was then analyzed using the Celigo Image Cytometer (Revvity) at time zero, and then for monitoring cell repopulation at 4 days later.

### Cell proliferation assay

Cells were seeded in triplicate in a 96-well plate. The day of seeding, the cells were treated with a low dose of KCNMA1’s modulators as described in the figure legend. Seven days post-treatment, cells were stained with Hoechst 33342 for 15 min. Fluorescence was then analyzed using the Celigo Image Cytometer (Revvity).

### Immunocytochemistry

The human cell lines ovc316 and RPE-1 were seeded on glass coverslips in 6 cm diameter dishes, grown to sub-confluency, then formalin-fixed, and the KCNMA1 protein was detected using the sensitive, previously applied indirect immunoperoxidase staining method with chromogen metal enhancement and without nuclear counterstaining [80]. The primary anti-KCNMA1 antibody (Anti-Maxi K/BK, Merck, ZRB2180) was used at a 1:1000 dilution. In the negative control staining, the primary antibody was replaced by non-immune serum at 1:1000 dilution. After multiple washes, the Vectastain Elite kit (Vector Laboratories) was employed as the secondary reagent to detect the bound primary antibody. Representative examples of the obtained results are shown in Supplementary Fig. 6, and the interpretation of the subcellular localization is explained in the main text (see the Results).

### Data analysis

GEPIA3 (https://gepia3.bioinfoliu.com) was used with default settings to perform correlation analysis of the OV TCGA data set for PROM1 and KCNMA1 [37]. cBioPortal (https://www.cbioportal.org) [57–59] was used to analyze dysregulations of selected ion channels in ovarian cancer using TCGA cancer genomic datasets [55, 56]. Other data were plotted using the following software tools: GraphPad Prism 9 (GraphPad software, version 9.5.1), RStudio (Posit Software, version 2023.06.0) and TIBCO Spotfire (Cloud Software Group, Inc., version 12.0.3.77).

Statistical analyzes were performed on medians of raw (non-normalized) data using RStudio. Culture fluorescence median intensity-centered (normalized) values were processed and analyzed as log2 fold changes for Fig. 3. Data are shown as mean ± SD, unless stated otherwise. Linear mixed-effects models were fitted using the lmer function (R package *lme4*) [81], with treatment condition and cell variant as fixed effects and experimental repetition as a random effect. In selected analyzes, estimated marginal means were computed using the *emmeans* package, and pairwise comparisons were performed with Tukey correction for multiple testing. The analysis of combined calcium and potassium signals (Fig. 4a, b, and Supplementary Fig. 6a, b), E-cadherin (Supplementary Fig. 4b), and Mitotracker DeepRed (Supplementary Fig. 6c) were performed on individual single-cell data due to alterations in distribution shape and subpopulation behavior that weren’t captured by summary statistics. Including replicate structure in the statistics allowed us to preserve statistical power and sensitivity. Descriptive statistics in Supplementary Table 1 were calculated using GraphPad Prism. The sample size (*N*) refers to the number of independent biological replicates.

### Chemicals

BMS-191011 (MedChem Express, HY-108593), Tetrandrine (Selleck Chemicals, S2403), Calcium chloride dihydrate (Sigma-Aldrich, C3306), Potassium chloride (AppliChem, A3582), YM-58483 (CRACi, Abcam, AB144413), Penitrem A (Focus Biomolecules, 10-2099), Cisplatin (NOMECO, 598049), Olaparib (Selleckchem, S1060).

## Supplementary information

Supplementary Figures

Supplementary Table 1

Supplementary Table 2

Supplementary Data

## Data Availability

Cell lines and data sets generated during this project will be available upon request from the corresponding authors.
