# Supplementary material for: Dynamic calcium–potassium balancing by KCNMA1 preserves the epithelial/mesenchymal hybrid state and modulates therapy response in ovarian cancer
Source: Cell Death Discov. 2026 Jun 4;12:331. doi: 10.1038/s41420-026-03189-6 (PMC13454608; doi:10.1038/s41420-026-03189-6)
Supplement: Supplementary file 1 [file 41420_2026_3189_MOESM2_ESM.pdf]

Supplement Table 1: Statistics based on quantitative image-based cytometry

Descriptive statistics of normalized calcium levels across CD133-defined cell states

CD133 level	low	intermediate	high
Number of values	9	9	9
Minimum	-0,0855	-0,1143	-0,8077
Maximum	0,2195	0,05992	0,3429
Range	0,305	0,1742	1,151
Mean	0,00126	2,3E-05	-0,0336
Std. Deviation	0,09573	0,05873	0,3336
Std. Error of Mean	0,03191	0,01958	0,1112

E-cadherin

75-100 %

contrast	estimate	SE	df	t.ratio	p.value
Control Mock - KCNMA1 Mock	-0,06381	0,048485	10	-1,31605	0,31097
Control Mock - (Control BMS-191011)	0,084456	0,048485	10	1,741893	0,210265
Control Mock - (KCNMA1 BMS-191011)	0,039219	0,048485	10	0,808892	0,468642
Control Mock - Control Tetrandrine	-0,02304	0,048485	10	-0,47525	0,644818
Control Mock - KCNMA1 Tetrandrine	-0,12827	0,048485	10	-2,64544	0,091891
KCNMA1 Mock - (Control BMS-191011)	0,148265	0,048485	10	3,057939	0,060435
KCNMA1 Mock - (KCNMA1 BMS-191011)	0,103028	0,048485	10	2,124938	0,127557
KCNMA1 Mock - Control Tetrandrine	0,040766	0,048485	10	0,840794	0,468642
KCNMA1 Mock - KCNMA1 Tetrandrine	-0,06446	0,048485	10	-1,32939	0,31097
(Control BMS-191011) - (KCNMA1 BMS-191011)	-0,04524	0,048485	10	-0,933	0,465998
(Control BMS-191011) - Control Tetrandrine	-0,1075	0,048485	10	-2,21714	0,127557
(Control BMS-191011) - KCNMA1 Tetrandrine	-0,21272	0,048485	10	-4,38733	0,020426
(KCNMA1 BMS-191011) - Control Tetrandrine	-0,06226	0,048485	10	-1,28414	0,31097
(KCNMA1 BMS-191011) - KCNMA1 Tetrandrine	-0,16748	0,048485	10	-3,45433	0,046359
Control Tetrandrine - KCNMA1 Tetrandrine	-0,10522	0,048485	10	-2,17019	0,127557

50-75 %

contrast	estimate	SE	df	t.ratio	p.value
Control Mock - KCNMA1 Mock	0,077581	0,023446	10	3,308913	0,019734
Control Mock - (Control BMS-191011)	0,074912	0,023446	10	3,195073	0,02051
Control Mock - (KCNMA1 BMS-191011)	0,081753	0,023446	10	3,486845	0,017561
Control Mock - Control Tetrandrine	-0,03763	0,023446	10	-1,60504	0,23261
Control Mock - KCNMA1 Tetrandrine	0,057213	0,023446	10	2,4402	0,06532
KCNMA1 Mock - (Control BMS-191011)	-0,00267	0,023446	10	-0,11384	0,911617
KCNMA1 Mock - (KCNMA1 BMS-191011)	0,004172	0,023446	10	0,177931	0,911617
KCNMA1 Mock - Control Tetrandrine	-0,11521	0,023446	10	-4,91395	0,003618
KCNMA1 Mock - KCNMA1 Tetrandrine	-0,02037	0,023446	10	-0,86871	0,552778
(Control BMS-191011) - (KCNMA1 BMS-19101	0,006841	0,023446	10	0,291772	0,895874
(Control BMS-191011) - Control Tetrandrine	-0,11254	0,023446	10	-4,80011	0,003618
(Control BMS-191011) - KCNMA1 Tetrandrine	-0,0177	0,023446	10	-0,75487	0,584663
(KCNMA1 BMS-191011) - Control Tetrandrine	-0,11938	0,023446	10	-5,09189	0,003618
(KCNMA1 BMS-191011) - KCNMA1 Tetrandrin	-0,02454	0,023446	10	-1,04665	0,479853
Control Tetrandrine - KCNMA1 Tetrandrine	0,094845	0,023446	10	4,045241	0,00878

25-50 %

contrast	estimate	SE	df	t.ratio	p.value
Control Mo	0,108007	0,011322	10	9,539803	9,16E-06
Control Mo	0,034289	0,011322	10	3,028575	0,015885
Control Mo	0,062674	0,011322	10	5,535758	0,000467
Control Mo	-0,0022	0,011322	10	-0,1939	0,850135
Control Mo	0,12	0,011322	10	10,5991	6,98E-06
KCNMA1 M	-0,07372	0,011322	10	-6,51123	0,00017
KCNMA1 M	-0,04533	0,011322	10	-4,00404	0,003753
KCNMA1 M	-0,1102	0,011322	10	-9,7337	9,16E-06
KCNMA1 M	0,011993	0,011322	10	1,059296	0,336836
(Control BM	0,028386	0,011322	10	2,507183	0,035841
(Control BM	-0,03648	0,011322	10	-3,22248	0,012459
(Control BM	0,085711	0,011322	10	7,570524	5,71E-05
(KCNMA1 B	-0,06487	0,011322	10	-5,72966	0,000408
(KCNMA1 B	0,057326	0,011322	10	5,063341	0,000816
Control Tet	0,122195	0,011322	10	10,793	6,98E-06

0-25 %

contrast	estimate	SE	df	t.ratio	p.value
Control Mo	0,088102	0,079551	10	1,107491	0,441013
Control Mo	-0,17353	0,079551	10	-2,18135	0,115976
Control Mo	-0,10186	0,079551	10	-1,28045	0,382151
Control Mo	0,072919	0,079551	10	0,916636	0,448641
Control Mo	0,154886	0,079551	10	1,947008	0,150272
KCNMA1 M	-0,26163	0,079551	10	-3,28884	0,042335
KCNMA1 M	-0,18996	0,079551	10	-2,38794	0,114278
KCNMA1 M	-0,01518	0,079551	10	-0,19086	0,852458
KCNMA1 M	0,066784	0,079551	10	0,839517	0,450853
(Control BM	0,071668	0,079551	10	0,900902	0,448641
(Control BM	0,246448	0,079551	10	3,097983	0,042335
(Control BM	0,328415	0,079551	10	4,128355	0,030743
(KCNMA1 B	0,17478	0,079551	10	2,197081	0,115976
(KCNMA1 B	0,256747	0,079551	10	3,227453	0,042335
Control Tet	0,081967	0,079551	10	1,030372	0,446062

Calcium

75-100 %

contrast	estimate	SE	df	t.ratio	p.value
Mock - (BMS-191011 5)	0,042101	0,037979	4	1,108545	0,329788
Mock - Tetrandrine 1	-0,06355	0,037979	4	-1,6732	0,254405
(BMS-191011 5) - Tetrandrine 1	-0,10565	0,037979	4	-2,78174	0,149189

50-75 %

contrast	estimate	SE	df	t.ratio	p.value
Mock - (BMS-191011 5)	0,020274	0,014487	4	1,399484	0,259794
Mock - Tetrandrine 1	-0,019	0,014487	4	-1,31185	0,259794
(BMS-191011 5) - Tetrandrine 1	-0,03928	0,014487	4	-2,71133	0,160391

25-50 %

contrast	estimate	SE	df	t.ratio	p.value
Mock - (BM	0,010324	0,008696	4	1,187178	0,300841
Mock - Tetr	0,031842	0,008696	4	3,661623	0,064648
(BMS-1910	0,021518	0,008696	4	2,474445	0,102928

0-25 %

contrast	estimate	SE	df	t.ratio	p.value
Mock - (BM	-0,06932	0,042453	4	-1,63284	0,208434
Mock - Tetr	0,063604	0,042453	4	1,498237	0,208434
(BMS-1910	0,132922	0,042453	4	3,131076	0,105446

Potassium

75-100 %

contrast	estimate	SE	df	t.ratio	p.value
Mock - (BMS-191011 5)	0,042143	0,040332	4	1,044916	0,355048
Mock - Tetrandrine 1	-0,17653	0,040332	4	-4,37704	0,017854
(BMS-191011 5) - Tetrandrine 1	-0,21868	0,040332	4	-5,42195	0,016829

50-75 %

contrast	estimate	SE	df	t.ratio	p.value
Mock - (BMS-191011 5)	0,024664	0,036055	4	0,684065	0,71015
Mock - Tetrandrine 1	-0,01439	0,036055	4	-0,39919	0,71015
(BMS-191011 5) - Tetrandrine 1	-0,03906	0,036055	4	-1,08325	0,71015

25-50 %

contrast	estimate	SE	df	t.ratio	p.value
Mock - (BM	0,012185	0,014289	4	0,852759	0,441845
Mock - Tetr	0,074893	0,014289	4	5,241437	0,017692
(BMS-1910	0,062709	0,014289	4	4,388679	0,017692

0-25 %

contrast	estimate	SE	df	t.ratio	p.value
Mock - (BM	-0,07796	0,07976	4	-0,97743	0,383702
Mock - Tetr	0,148834	0,07976	4	1,866019	0,20318
(BMS-1910	0,226793	0,07976	4	2,843445	0,140113

Log(Ca x K + 1)

75-100 %

contrast	estimate	SE	df	t.ratio	p.value
Mock - (BMS-191011 5)	0,044973	0,037539	4	1,198032	0,297036
Mock - Tetrandrine 1	-0,13101	0,037539	4	-3,48998	0,037685
(BMS-191011 5) - Tetrandrine 1	-0,17598	0,037539	4	-4,68801	0,028175

50-75 %

contrast	estimate	SE	df	t.ratio	p.value
Mock - (BMS-191011 5)	0,02165	0,024389	4	0,887702	0,424856
Mock - Tetrandrine 1	-0,02731	0,024389	4	-1,11995	0,424856
(BMS-191011 5) - Tetrandrine 1	-0,04896	0,024389	4	-2,00765	0,345321

25-50 %

contrast	estimate	SE	df	t.ratio	p.value
Mock - (BM	0,014614	0,012124	4	1,205329	0,294504
Mock - Tetr	0,043328	0,012124	4	3,573692	0,069896
(BMS-1910	0,028714	0,012124	4	2,368363	0,115437

0-25 %

contrast	estimate	SE	df	t.ratio	p.value
Mock - (BM	-0,07999	0,064749	4	-1,23546	0,284262
Mock - Tetr	0,124169	0,064749	4	1,917704	0,191413
(BMS-1910	0,204163	0,064749	4	3,15316	0,103228

Mitotracker

75-100 %

contrast	estimate	SE	df	t.ratio	p.value
Mock - (BMS-204352)	0,015031	0,02899	4	0,518474	0,631488
Mock - Tetrandrine	-0,11915	0,02899	4	-4,10993	0,0221
(BMS-204352) - Tetrandrine	-0,13418	0,02899	4	-4,6284	0,0221

50-75 %

contrast	estimate	SE	df	t.ratio	p.value
Mock - (BMS-204352)	0,011054	0,016481	4	0,670751	0,539119
Mock - Tetrandrine	0,02871	0,016481	4	1,742051	0,469388
(BMS-204352) - Tetrandrine	0,017656	0,016481	4	1,071299	0,516556

25-50 %

contrast	estimate	SE	df	t.ratio	p.value
Mock - (BM	0,002247	0,018265	4	0,123028	0,908019
Mock - Tetr	0,078854	0,018265	4	4,317225	0,02064
(BMS-2043	0,076606	0,018265	4	4,194196	0,02064

0-25 %

contrast	estimate	SE	df	t.ratio	p.value
Mock - (BM	-0,02296	0,030969	4	-0,74125	0,499698
Mock - Tetr	0,08734	0,030969	4	2,820207	0,071727
(BMS-2043	0,110296	0,030969	4	3,56146	0,070666
