# Supplementary material for: Dynamic calcium–potassium balancing by KCNMA1 preserves the epithelial/mesenchymal hybrid state and modulates therapy response in ovarian cancer
Source: Cell Death Discov. 2026 Jun 4;12:331. doi: 10.1038/s41420-026-03189-6 (PMC13454608; doi:10.1038/s41420-026-03189-6)
Supplement: Supplementary file 2 [file 41420_2026_3189_MOESM3_ESM.pdf]

Supplement Table 2: Overview table for results of proliferation assays

ovc316^{Overexpressi}	Treatment	3D: sphere formation	2D: Scratch regrowth	2D: Low density
ovc316^{Control*}	DMSO	reference	reference	reference
ovc316^{Control*}	Tetrandrine	enhanced	unchanged	suppressed
ovc316^{Control*}	BMS-191011	unchanged	faster	moderately enhanced
ovc316^{KCNMA1}	DMSO	enhanced	slower	/
ovc316^{KCNMA1}	Tetrandrine	enhanced**	moderately faster**	/
ovc316^{KCNMA1}	BMS-191011	suppressed**	unchanged**	/
HOEC	DMSO	/	/	reference
HOEC	Tetrandrine	/	/	unchanged
HOEC	BMS-191011	/	/	unchanged

* ovc316 parental or overepxressing control vector

** compared to KCNMA1 overexpressing cells
