# Supplementary material for: Dynamic calcium–potassium balancing by KCNMA1 preserves the epithelial/mesenchymal hybrid state and modulates therapy response in ovarian cancer
Source: Cell Death Discov. 2026 Jun 4;12:331. doi: 10.1038/s41420-026-03189-6 (PMC13454608; doi:10.1038/s41420-026-03189-6)
Supplement: Supplementary file 3 [file 41420_2026_3189_MOESM1_ESM.docx]

**Supplementary Fig. 1: KCNMA1 blocking moderately elevates total surface E-cadherin presence.** The bar graph shows the effect of KCNMA1 modulation on E-cadherin expression in ovc316 cells. Cells were treated for four days with 10μM doses of KCNMA1 modulators BMS-191011 and tetrandrine and then analyzed by flow cytometer (N = 4, DF = 6).

**Supplementary Fig. 2: KCNMA1 activity modulation alters differentiation status and phenotype.** (A) The density plot showing CD133 against E-cadherin in ovc316 cells. The cells were treated with DMSO, BMS-191011 (10μM), tetrandrine (5μM), tetrandrine (5μM)/DMSO, and tetrandrine (5μM)/BMS-191011 (10μM) daily for four days (N = 3). (B, C) The bar graph represents median E-cadherin in cells treated with DMSO, BMS-191011 (10μM), tetrandrine (5μM), tetrandrine (5μM)/DMSO, and tetrandrine (5μM)/BMS-191011 (10μM) daily for four days, and then analyzed by flow cytometer. (N = 3, DF 8).

**Supplementary Fig. 3: KCNMA1 modulation alters differentiation status and phenotype of ovarian cancer cell lines.** (A) Western blot visualization of KCNMA1 expression in different cell lines (HOEC hTERT, OVCAR4, Skov-3 and UWB1.289), (N = 3). (B-F) Western blot visualizations of total CD133 in HOEC hTERT, OVCAR4, Skov-3 and UWB1.289 cell lines after treatment with 10μM KCNMA1 modulators, BMS-191011 and tetrandrine. The data were further accompanied by flow cytometry density plots evaluating CD133 and E-cadherin. Additionally, median expression levels of CD133, and E-cadherin were plotted using box plot. HOEC hTERT: N = 3, DF = 4; OVCAR4: N = 3, DF = 4 (CD133), 6 (E-cadherin); Skov-3: N = 3, DF = 4; UWB1.289: N = 3, DF = 4 (Note different antibodies were used for western blot and flow cytometry).

Abbreviation: AF488 Alexa Fluor 488, HOEC Human ovarian epithelial cells, kDa kilodalton

**Supplementary Fig. 4: KCNMA1 overexpressing ovc316 cells are responsive to tetrandrine in similar extend.** (A) Western blot verification of KCNMA1 overexpression (N = 3). (B) 100% stacked bar graph shows differences in E-cadherin fluorescent intensity between treatments relative to Control Mock. The bars contain proportion of cells ± SD in subpopulation (significant bars are with red stroke). Cells were treated for four days with 10μM doses of KCNMA1 modulators (N = 3, *emmeans*, DF = 10).

Abbreviations: kDa Kilodalton

**Supplementary Fig. 5: KCNMA1 overexpression and basal cellular state affect cell growth.** (A) Images depicting sphere formation assay of control and KCNMA1 overexpressing cells treated with low doses of channel activator (BMS-191011, 1μM) and blocker (tetrandrine, 1μM) for 6 weeks (N = 3). (B) Graphical visualization of regrown area in scratch assay of control and KCNMA1 overexpressing cells in scratch assay. Cells were treated with BMS-191011 and tetrandrine (2.5 and 5μM) for 7 days (N = 4, DF = 6).

**Supplementary Fig. 6: Changes in ions and mitochondrial activity by KCNMA1 manipulation in ovc316 are dependent on the basal cell state**. (A, B) 100% stacked bar graphs show differences in PBFI AM (Potassium) and Oregon Green 488 BAPTA-1 AM (Calcium) fluorescent intensities between treatments relative to Mock. The bars contain proportion of cells ± SD in subpopulation (significant bars are with red stroke). Cells were treated for four days with 5μM (BMS-191011) and 1μM (tetrandrine) doses of KCNMA1 modulators (N = 3, *emmeans*, DF = 4). (C) 100% stacked bar graph of data from ScanR microscopy-based cytometry analyzing mitochondrial membrane potential (Mitotracker DeepRed). The graph shows differences in treatments relative to Mock. The bars contain proportion of cells ± SD in subpopulation (significant bars are with red stroke). Ovc316 cell were treated by BMS-191011 and tetrandrine (10μM) for four days. Scale bar 40µm (N = 3, *emmeans*, DF = 4).

Abbreviations: A.U. Arbitrary Unit, MT-DR Mitotracker DeepRed

**Supplementary Fig. 6: Immunocytochemical detection of KCNMA1 in human cell lines.** Upper left: Representative image of cultured ovc316 cells. Upper right: Magnified section of ovc316 cells from the upper left panel. Arrow points to the KCNMA1 stained cell membrane. Lower left: Representative image of KCNMA1 staining pattern in cultured RPE-1 hTERT cells. Lower right: Negative control staining of the RPE-1 hTERT cells (see Methods for details). Scale bar: 50 µm.

**Supplementary Fig. 8: CD133, CD44 and E-cadherin are altered by direct manipulation of calcium.** A) The flow cytometry density plot evaluates CD44 and E-cadherin and complements histograms visualizing CD133. Cells were treated for four days with BMS-191011 (10μM), tetrandrine (10μM), calcium (5mM), potassium (5mM), CRACi (1μM). Calcium, potassium and CRACi were further combined with tetrandrine. The cells were then analyzed by flow cytometer (N = 3). (B, C) The bar graph visualizations of CD133 and E-cadherin (both E-cadherin-high/CD44-high quadrant) expression in ovc316 treated for four days with BMS-191011 (10μM), tetrandrine (10μM), calcium (5mM), potassium (5mM), CRACi (1μM). Calcium, potassium and CRACi were further combined with tetrandrine. The cells were then analyzed by flow cytometer (N = 3, DF = 16). (D) The flow cytometry density plot evaluates CD133 and E-cadherin. Cells were treated for four days with different ratios of calcium and potassium and then analyzed by flow cytometer (N = 4).

Abbreviations: AF488 Alexa fluor 488

**Supplementary Fig. 9: KCNMA1 and related channels are genetically altered in 59% of high-grade serous ovarian tumors.** cBioPortal derived graph visualizes genetic profile of selected ion channel genes in ovarian cancer patients (TCGA data sets, N=1074). Abbreviations: CNA copy number alterations, mut. mutations, SV structural variants

**Supplementary Fig. 10: Example of gating strategy applied to flow cytometry data.** Each dataset was sequentially gated to exclude debris, doublets, and dead cells. The remaining live cells were further gated based on the main population in the control sample. Unstrained control was consistently located in the lower left quadrant.
