# Supplementary material for: Dynamic calcium–potassium balancing by KCNMA1 preserves the epithelial/mesenchymal hybrid state and modulates therapy response in ovarian cancer
Source: Cell Death Discov. 2026 Jun 4;12:331. doi: 10.1038/s41420-026-03189-6 (PMC13454608; doi:10.1038/s41420-026-03189-6)
Supplement: Supplementary file 4 [file 41420_2026_3189_MOESM4_ESM.docx]

**Supplementary Data 1:** Uncropped western blot images corresponding to Fig. 1.

**Supplementary Data 2:** Uncropped western blot images corresponding to Supplementary Fig. 3.

**Supplementary Data 3:** Uncropped western blot images corresponding to Supplementary Fig. 4.

**Supplementary Data 4:** Uncropped western blot images corresponding to Fig. 2.
